# 9-(4-Bromo­but­yl)-9*H*-carbazole

**DOI:** 10.1107/S1600536812010987

**Published:** 2012-03-17

**Authors:** Qing-Peng Wang, Juan-Juan Chang, Hui-Zhen Zhang, Jing-Song Lv, Cheng-He Zhou

**Affiliations:** a Laboratory of Bioorganic & Medicinal Chemistry, School of Chemistry and Chemical Engineering, Southwest University, Chongqing 400715, People’s Republic of China

## Abstract

In the title compound, C_16_H_16_BrN, the bromo­butyl group lies on one side of the carbazole ring plane and has a zigzag shape. The dihedral angle between the two benzene rings is 0.55°. In the crystal, mol­ecules are connected by van der Waals inter­actions.

## Related literature

For charge-transport properties and π-conjugated systems in carbazoles, see: Zhang *et al.* (2010*a* ▶). For the bioactivity of carbazole derivatives, see: Yan *et al.* (2011 ▶). For the synthesis of the title compound, see: Zhang *et al.* (2010*b* ▶).
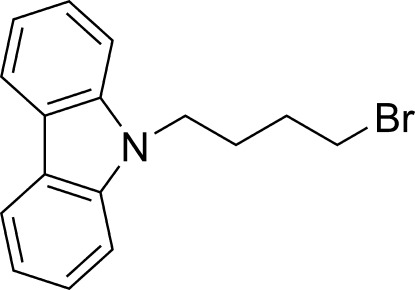


## Experimental

### 

#### Crystal data

C_16_H_16_BrN
*M* *_r_* = 302.20
Orthorhombic, 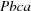

*a* = 7.696 (3) Å
*b* = 22.658 (8) Å
*c* = 16.030 (6) Å
*V* = 2795.3 (18) Å^3^
*Z* = 8
Mo *K*α radiation
μ = 2.92 mm^−1^
*T* = 296 K
0.35 × 0.33 × 0.32 mm

#### Data collection

Bruker SMART CCD diffractometer
Absorption correction: multi-scan (*SADABS*; Sheldrick, 1996 ▶) *T*_min_ = 0.428, *T*_max_ = 0.455
13981 measured reflections
2460 independent reflections
1252 reflections with *I* > 2σ(*I*)
*R*_int_ = 0.094

#### Refinement

*R*[*F* ^2^ > 2σ(*F* ^2^)] = 0.050
*wR*(*F* ^2^) = 0.145
*S* = 0.97
2460 reflections
163 parameters
H-atom parameters constrained
Δρ_max_ = 0.35 e Å^−3^
Δρ_min_ = −0.42 e Å^−3^

### 

Data collection: *SMART* (Bruker, 2001 ▶); cell refinement: *SAINT-Plus* (Bruker, 2001 ▶); data reduction: *SAINT-Plus*; program(s) used to solve structure: *SHELXS97* (Sheldrick, 2008 ▶); program(s) used to refine structure: *SHELXL97* (Sheldrick, 2008 ▶); molecular graphics: *SHELXTL* (Sheldrick, 2008 ▶); software used to prepare material for publication: *SHELXTL*.

## Supplementary Material

Crystal structure: contains datablock(s) global, I. DOI: 10.1107/S1600536812010987/rk2343sup1.cif

Structure factors: contains datablock(s) I. DOI: 10.1107/S1600536812010987/rk2343Isup2.hkl

Supplementary material file. DOI: 10.1107/S1600536812010987/rk2343Isup3.cml

Additional supplementary materials:  crystallographic information; 3D view; checkCIF report

## Figures and Tables

**Fig. 1.**
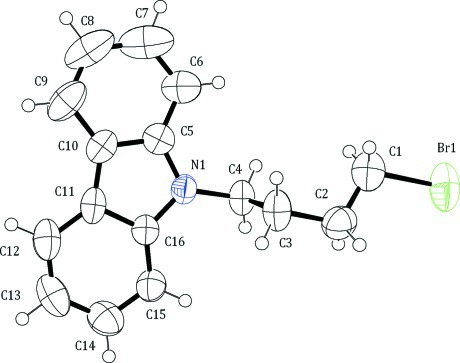
The molecular structure of I, showing the atom-numbering scheme. Displacement ellipsoids are drawn at the 50% probability level. H atoms are presented as a small spheres of arbitrary radius.

## supplementary crystallographic information

### Comment

Carbazole and its derivatives as an important type of aromatic compounds are being actively investigated for their special structural characteristics with desirable electronic charge-transport properties and π-conjugated system (Zhang *et al.*, 2010*a*). Large amount of bioactive carbazole derivatives have been reported to exert diverse biological activities such as antitumor, antimicrobial, antihistaminic, antioxidative, anti-inflammatory ones and so on (Yan *et al.*, 2011). Our interest is to develop novel carbazole compounds as medicinal agents. Herein, the molecular structure of the title compound, I, is reported.

The X-ray analysis of I shows that the carbon C4 and carbazole moiety (N1/C5–C16) belong to the same plane. The bromobutyl moiety lies in the same side of the carbon plane.

### Experimental

The title compound was synthesized according to the procedure of Zhang *et al.* (2010*b*). Single crystals were grown by slow evaporation of a solution of I in CHCl_3_ at room temperature.

### Refinement

H atoms were placed at calculated positions with C—H = 0.93Å (aromatic) and 0.97Å (methylene). The *U*_iso_(H) = 1.2*U*_eq_(C).

### Crystal data

**Table:**

C_16_H_16_BrN	*F*(000) = 1232
*M**_r_* = 302.20	*D*_x_ = 1.436 Mg m^−^^3^
Orthorhombic, *P**b**c**a*	Mo *K*α radiation, λ = 0.71073 Å
Hall symbol: -P 2ac 2ab	Cell parameters from 1683 reflections
*a* = 7.696 (3) Å	θ = 2.2–20.5°
*b* = 22.658 (8) Å	µ = 2.92 mm^−^^1^
*c* = 16.030 (6) Å	*T* = 296 K
*V* = 2795.3 (18) Å^3^	Block, colourless
*Z* = 8	0.35 × 0.33 × 0.32 mm

### Data collection

**Table:**

Bruker SMART CCD diffractometer	2460 independent reflections
Radiation source: fine-focus sealed tube	1252 reflections with *I* > 2σ(*I*)
Graphite monochromator	*R*_int_ = 0.094
φ and ω scans	θ_max_ = 25.0°, θ_min_ = 2.2°
Absorption correction: multi-scan (*SADABS*; Sheldrick, 1996)	*h* = −9→9
*T*_min_ = 0.428, *T*_max_ = 0.455	*k* = −26→23
13981 measured reflections	*l* = −19→18

### Refinement

**Table:**

Refinement on *F*^2^	Primary atom site location: structure-invariant direct methods
Least-squares matrix: full	Secondary atom site location: difference Fourier map
*R*[*F*^2^ > 2σ(*F*^2^)] = 0.050	Hydrogen site location: inferred from neighbouring sites
*wR*(*F*^2^) = 0.145	H-atom parameters constrained
*S* = 0.97	*w* = 1/[σ^2^(*F*_o_^2^) + (0.0728*P*)^2^] where *P* = (*F*_o_^2^ + 2*F*_c_^2^)/3
2460 reflections	(Δ/σ)_max_ < 0.001
163 parameters	Δρ_max_ = 0.35 e Å^−^^3^
0 restraints	Δρ_min_ = −0.42 e Å^−^^3^

### Special details

**Table:**

Geometry. All s.u.'s (except the s.u. in the dihedral angle between two l.s. planes) are estimated using the full covariance matrix. The cell s.u.'s are taken into account individually in the estimation of s.u.'s in distances, angles and torsion angles; correlations between s.u.'s in cell parameters are only used when they are defined by crystal symmetry. An approximate (isotropic) treatment of cell s.u.'s is used for estimating s.u.'s involving l.s. planes.
Refinement. Refinement of *F*^2^ against ALL reflections. The weighted *R*-factor *wR* and goodness of fit *S* are based on *F*^2^, conventional *R*-factors *R* are based on *F*, with *F* set to zero for negative *F*^2^. The threshold expression of *F*^2^ > σ(*F*^2^) is used only for calculating *R*-factors(gt) *etc*. and is not relevant to the choice of reflections for refinement. *R*-factors based on *F*^2^ are statistically about twice as large as those based on *F*, and *R*-factors based on ALL data will be even larger.

### Fractional atomic coordinates and isotropic or equivalent isotropic displacement parameters (Å^2^)

**Table:**

	*x*	*y*	*z*	*U*_iso_*/*U*_eq_	
Br1	1.17750 (8)	0.51102 (3)	0.14901 (4)	0.0961 (4)	
N1	0.4563 (4)	0.63416 (15)	0.0682 (2)	0.0503 (9)	
C1	0.9838 (6)	0.5506 (2)	0.0916 (3)	0.0804 (16)	
H1A	1.0214	0.5640	0.0371	0.097*	
H1B	0.8887	0.5230	0.0840	0.097*	
C2	0.9256 (7)	0.6005 (2)	0.1406 (3)	0.0728 (15)	
H2A	1.0216	0.6275	0.1495	0.087*	
H2B	0.8853	0.5869	0.1946	0.087*	
C3	0.7738 (6)	0.6334 (2)	0.0939 (3)	0.0710 (16)	
H3A	0.7660	0.6736	0.1143	0.085*	
H3B	0.7995	0.6349	0.0347	0.085*	
C4	0.6020 (6)	0.60279 (19)	0.1069 (3)	0.0589 (12)	
H4A	0.6086	0.5633	0.0837	0.071*	
H4B	0.5804	0.5991	0.1662	0.071*	
C5	0.3858 (6)	0.6217 (2)	−0.0087 (3)	0.0534 (12)	
C6	0.4334 (8)	0.5788 (2)	−0.0656 (3)	0.0744 (15)	
H6	0.5249	0.5531	−0.0549	0.089*	
C7	0.3397 (11)	0.5757 (3)	−0.1393 (4)	0.107 (2)	
H7	0.3692	0.5475	−0.1791	0.128*	
C8	0.2017 (10)	0.6140 (4)	−0.1546 (4)	0.106 (3)	
H8	0.1403	0.6107	−0.2044	0.127*	
C9	0.1550 (7)	0.6562 (3)	−0.0986 (4)	0.0820 (17)	
H9	0.0626	0.6815	−0.1095	0.098*	
C10	0.2482 (6)	0.6607 (2)	−0.0248 (3)	0.0546 (12)	
C11	0.2370 (5)	0.6987 (2)	0.0468 (3)	0.0517 (12)	
C12	0.1309 (6)	0.7458 (2)	0.0690 (4)	0.0684 (15)	
H12	0.0438	0.7587	0.0331	0.082*	
C13	0.1557 (7)	0.7729 (3)	0.1436 (4)	0.0810 (17)	
H13	0.0852	0.8046	0.1584	0.097*	
C14	0.2847 (7)	0.7542 (2)	0.1985 (4)	0.0744 (16)	
H14	0.2990	0.7734	0.2493	0.089*	
C15	0.3906 (6)	0.7079 (2)	0.1782 (3)	0.0576 (12)	
H15	0.4762	0.6952	0.2150	0.069*	
C16	0.3682 (5)	0.68078 (19)	0.1028 (3)	0.0462 (11)	

### Atomic displacement parameters (Å^2^)

**Table:**

	*U*^11^	*U*^22^	*U*^33^	*U*^12^	*U*^13^	*U*^23^
Br1	0.0657 (4)	0.1089 (6)	0.1138 (6)	0.0220 (3)	0.0026 (3)	0.0376 (4)
N1	0.046 (2)	0.051 (2)	0.054 (3)	0.0042 (18)	−0.0053 (18)	0.0027 (19)
C1	0.068 (4)	0.086 (4)	0.087 (4)	0.003 (3)	0.000 (3)	0.013 (3)
C2	0.062 (3)	0.089 (4)	0.068 (4)	−0.010 (3)	0.001 (3)	−0.007 (3)
C3	0.049 (3)	0.070 (3)	0.094 (5)	0.004 (2)	0.000 (3)	0.017 (3)
C4	0.046 (3)	0.059 (3)	0.072 (3)	0.006 (2)	−0.002 (3)	0.011 (3)
C5	0.055 (3)	0.047 (3)	0.058 (3)	−0.011 (2)	0.008 (3)	0.006 (3)
C6	0.101 (4)	0.060 (3)	0.062 (4)	−0.010 (3)	0.003 (3)	0.004 (3)
C7	0.171 (8)	0.082 (5)	0.067 (5)	−0.049 (5)	0.010 (5)	−0.003 (4)
C8	0.151 (7)	0.095 (5)	0.072 (5)	−0.050 (5)	−0.043 (5)	0.022 (4)
C9	0.090 (4)	0.085 (4)	0.071 (4)	−0.030 (3)	−0.028 (4)	0.031 (4)
C10	0.050 (3)	0.060 (3)	0.054 (4)	−0.014 (2)	−0.003 (2)	0.015 (3)
C11	0.036 (2)	0.056 (3)	0.063 (4)	−0.002 (2)	0.003 (2)	0.024 (3)
C12	0.053 (3)	0.074 (4)	0.079 (4)	0.011 (3)	0.007 (3)	0.028 (3)
C13	0.075 (4)	0.071 (4)	0.097 (5)	0.020 (3)	0.030 (4)	0.013 (4)
C14	0.087 (4)	0.077 (4)	0.060 (4)	0.007 (3)	0.018 (3)	0.004 (3)
C15	0.056 (3)	0.067 (3)	0.050 (3)	0.003 (3)	0.003 (2)	0.009 (3)
C16	0.043 (3)	0.049 (3)	0.046 (3)	−0.002 (2)	0.006 (2)	0.010 (2)

### Geometric parameters (Å, º)

**Table:**

Br1—C1	1.968 (5)	C6—H6	0.9300
N1—C16	1.372 (5)	C7—C8	1.394 (10)
N1—C5	1.377 (5)	C7—H7	0.9300
N1—C4	1.465 (5)	C8—C9	1.361 (9)
C1—C2	1.447 (7)	C8—H8	0.9300
C1—H1A	0.9700	C9—C10	1.386 (7)
C1—H1B	0.9700	C9—H9	0.9300
C2—C3	1.575 (6)	C10—C11	1.437 (7)
C2—H2A	0.9700	C11—C12	1.391 (7)
C2—H2B	0.9700	C11—C16	1.411 (6)
C3—C4	1.507 (6)	C12—C13	1.358 (7)
C3—H3A	0.9700	C12—H12	0.9300
C3—H3B	0.9700	C13—C14	1.392 (8)
C4—H4A	0.9700	C13—H13	0.9300
C4—H4B	0.9700	C14—C15	1.367 (6)
C5—C6	1.382 (6)	C14—H14	0.9300
C5—C10	1.403 (6)	C15—C16	1.368 (6)
C6—C7	1.386 (8)	C15—H15	0.9300
			
C16—N1—C5	108.9 (4)	C7—C6—H6	121.3
C16—N1—C4	125.6 (4)	C6—C7—C8	121.0 (7)
C5—N1—C4	125.5 (4)	C6—C7—H7	119.5
C2—C1—Br1	109.7 (4)	C8—C7—H7	119.5
C2—C1—H1A	109.7	C9—C8—C7	121.5 (6)
Br1—C1—H1A	109.7	C9—C8—H8	119.2
C2—C1—H1B	109.7	C7—C8—H8	119.2
Br1—C1—H1B	109.7	C8—C9—C10	118.5 (6)
H1A—C1—H1B	108.2	C8—C9—H9	120.7
C1—C2—C3	110.0 (4)	C10—C9—H9	120.7
C1—C2—H2A	109.7	C9—C10—C5	120.1 (5)
C3—C2—H2A	109.7	C9—C10—C11	133.9 (5)
C1—C2—H2B	109.7	C5—C10—C11	106.0 (4)
C3—C2—H2B	109.7	C12—C11—C16	118.5 (5)
H2A—C2—H2B	108.2	C12—C11—C10	134.4 (5)
C4—C3—C2	111.6 (4)	C16—C11—C10	107.1 (4)
C4—C3—H3A	109.3	C13—C12—C11	119.4 (5)
C2—C3—H3A	109.3	C13—C12—H12	120.3
C4—C3—H3B	109.3	C11—C12—H12	120.3
C2—C3—H3B	109.3	C12—C13—C14	121.3 (5)
H3A—C3—H3B	108.0	C12—C13—H13	119.4
N1—C4—C3	113.0 (4)	C14—C13—H13	119.4
N1—C4—H4A	109.0	C15—C14—C13	120.6 (5)
C3—C4—H4A	109.0	C15—C14—H14	119.7
N1—C4—H4B	109.0	C13—C14—H14	119.7
C3—C4—H4B	109.0	C14—C15—C16	118.6 (4)
H4A—C4—H4B	107.8	C14—C15—H15	120.7
N1—C5—C6	129.1 (5)	C16—C15—H15	120.7
N1—C5—C10	109.5 (4)	C15—C16—N1	129.8 (4)
C6—C5—C10	121.4 (5)	C15—C16—C11	121.6 (4)
C5—C6—C7	117.4 (6)	N1—C16—C11	108.5 (4)
C5—C6—H6	121.3		
			
Br1—C1—C2—C3	178.5 (3)	C9—C10—C11—C12	−1.1 (9)
C1—C2—C3—C4	80.9 (5)	C5—C10—C11—C12	179.6 (5)
C16—N1—C4—C3	−83.0 (5)	C9—C10—C11—C16	179.2 (5)
C5—N1—C4—C3	96.9 (5)	C5—C10—C11—C16	−0.1 (5)
C2—C3—C4—N1	176.7 (4)	C16—C11—C12—C13	−0.3 (7)
C16—N1—C5—C6	179.6 (4)	C10—C11—C12—C13	180.0 (5)
C4—N1—C5—C6	−0.3 (7)	C11—C12—C13—C14	−0.3 (8)
C16—N1—C5—C10	−0.2 (5)	C12—C13—C14—C15	0.2 (8)
C4—N1—C5—C10	179.8 (4)	C13—C14—C15—C16	0.6 (7)
N1—C5—C6—C7	−180.0 (5)	C14—C15—C16—N1	179.9 (4)
C10—C5—C6—C7	−0.2 (7)	C14—C15—C16—C11	−1.2 (6)
C5—C6—C7—C8	−0.6 (8)	C5—N1—C16—C15	179.2 (4)
C6—C7—C8—C9	0.6 (10)	C4—N1—C16—C15	−0.9 (7)
C7—C8—C9—C10	0.1 (9)	C5—N1—C16—C11	0.2 (4)
C8—C9—C10—C5	−0.9 (7)	C4—N1—C16—C11	−179.9 (3)
C8—C9—C10—C11	179.9 (5)	C12—C11—C16—C15	1.1 (6)
N1—C5—C10—C9	−179.2 (4)	C10—C11—C16—C15	−179.1 (4)
C6—C5—C10—C9	0.9 (7)	C12—C11—C16—N1	−179.8 (3)
N1—C5—C10—C11	0.2 (5)	C10—C11—C16—N1	0.0 (5)
C6—C5—C10—C11	−179.6 (4)		
